# An autoantibody profile identified by human genome‐wide protein arrays in rheumatoid arthritis

**DOI:** 10.1002/mco2.679

**Published:** 2024-08-11

**Authors:** Xu Liu, Xiaoying Zhang, Yu‐Jian Kang, Fei Huang, Shuang Liu, Yixue Guo, Yingni Li, Changcheng Yin, Mingling Liu, Qimao Han, Qingwen Wang, Hua Ye, Haihong Yao, Chun Li, Jiahe Li, Wangzha Pingcuo, Yan Zhang, Yin Su, Ge Gao, Zhanguo Li, Xiaolin Sun

**Affiliations:** ^1^ Department of Rheumatology and Immunology Peking University People's Hospital & Beijing Key Laboratory for Rheumatism Mechanism and Immune Diagnosis (BZ0135) Beijing China; ^2^ Chongqing Key Laboratory of Intelligent Oncology for Breast Cancer Cancer Hospital School of Medicine Chongqing University Chongqing China; ^3^ General Medical Department Huazhong University of Science and Technology Union Shenzhen Hospital Shenzhen China; ^4^ Department of Rheumatology and Immunology First Affiliated Hospital of Kunming Medical University. Kunming China; ^5^ Beijing Protein Innovation B‐8, Airport Industrial Zone Beijing China; ^6^ Department of Rheumatology the First Affiliated Hospital of Guangzhou University of Chinese Medicine Guangzhou China; ^7^ Department of Rheumatology The First Affiliated Hospital of Heilongjiang University of Traditional Chinese Medicine. No.24 Heping Road Xiangfang District Harbin China; ^8^ Department of Rheumatism and Immunology Peking University Shenzhen Hospital Shenzhen China; ^9^ State Key Laboratory of Protein and Plant Gene Research, School of Life Sciences Biomedical Pioneering Innovative Center (BIOPIC) & Beijing Advanced Innovation Center for Genomics (ICG) Center for Bioinformatics (CBI) Peking University Beijing China

**Keywords:** anticitrullination protein antibody (ACPA), autoantibody, HuProt array, rheumatoid arthritis

## Abstract

Precise diagnostic biomarkers of anticitrullination protein antibody (ACPA)‐negative and early‐stage RA are still to be improved. We aimed to screen autoantibodies in ACPA‐negative patients and evaluated their diagnostic performance. The human genome‐wide protein arrays (HuProt arrays) were used to define specific autoantibodies from the sera of 182 RA patients and 261 disease and healthy controls. Statistical analysis was performed with SPSS 17.0. In Phase I study, 51 out of 19,275 recombinant proteins covering the whole human genome were selected. In Phase II validation study, anti‐ANAPC15 (anaphase promoting complex subunit 15) exhibited 41.8% sensitivity and 91.5% specificity among total RA patients. There were five autoantibodies increased in ACPA‐negative RA, including anti‐ANAPC15, anti‐LSP1, anti‐APBB1, anti‐parathymosin, and anti‐UBL7. Anti‐parathymosin showed the highest prevalence of 46.2% (*p* = 0.016) in ACPA‐negative early stage (<2 years) RA. To further improve the diagnostic efficacy, a prediction model was constructed with 44 autoantibodies. With increased threshold for RA calling, the specificity of the model is 90.8%, while the sensitivity is 66.1% (87.8% in ACPA‐positive RA and 23.8% in ACPA‐negative RA) in independent testing patients. Therefore, HuProt arrays identified RA‐associated autoantibodies that might become possible diagnostic markers, especially in early stage ACPA‐negative RA.

## Graphical abstract

HuProt array is a genome‐wide protein array suitable for high throughput unbiased analysis of autoantibodies against the entire human proteome without protein modifications.

We performed RA autoantibody screening in 182 RA patients and 261 controls with HuProt arrays to identify the nonmodified protein antibody profile of RA, especially in ACPA negative patients.

There were five autoantibodies increased in ACPA‐negative RA (anti‐ANAPC15, anti‐LSP1, anti‐APBB1, anti‐parathymosin, and anti‐UBL7). Among them, anti‐parathymosin showed the highest prevalence of 46.2% (*p* = 0.016) in ACPA‐negative and early stage (<2years) RA. Prediction model with 44 markers was also constructed with specificity of 90.8%, while the sensitivity is 66.1% (87.8% in ACPA‐positive RA patients and 23.8% in ACPA‐negative RA).

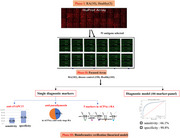

## 1 INTRODUCTION

Rheumatoid arthritis (RA) is an autoimmune disease featured by inflammatory synovitis and progressive joint destruction.^1^ Without timely diagnosis or early treatment, persistent joint swelling and inflammation leads to deformity and disability of joints, which brings about individual and social burdens. Pathogenesis of RA begins months to years when autoimmune response persists and development of polyarthritis. Therapeutic drugs are effective in the early stage and might gradually lose their drug response as the disease progresses. Autoantibodies especially anticitrullinated protein antibodies (ACPAs) have been proposed as specific biomarkers for RA diagnosis. ACPA was recruited by the 2010 European League against Rheumatism (EULAR)/American College of Rheumatology (ACR) classification criteria for RA, making ACPA the most specific diagnostic marker in RA. However, approximately one‐third of RA patients are ACPA negative.^2^ ACPA− and ACPA+ RA can be regarded as two disease subtypes with differences in pathogenesis.^3^ However, ACPA‐negative RA patients still suffer from disease progression due to delayed diagnosis and treatment.^4^ Besides ACPAs, other disease‐relevant molecules have also been found in RA serum and synovial fluid^5^, ^6^, ^7^, ^8^, ^9^ but the diagnostic efficacy is insufficient. Therefore, searching for autoantibodies in ACPA‐negative RA, especially in the early disease stage is of great importance.

Besides ACPAs, anticarbamylated antibodies have also been identified as specific markers for RA in recent years and applied in clinical diagnosis.^10^, ^11^ Other autoantibodies targeting a variety of proteins are also widely prevalent in RA patients,^12^, ^13^ while the autoantibody profiles and their clinical significance are not fully elucidated. Screening novel diagnostic autoantibodies is crucial for improvement of early diagnosis and timely treatment of RA, especially for ACPA‐negative RA.

Previous strategies for novel autoantibodies screening were built on selected sets of special target antigens. Identification of the diversity of autoimmune repertoire is dependent on the comprehensiveness of the applied antigen collection. The HuProt array is a genome‐wide protein array suitable for high throughput unbiased analysis of autoantibodies against human proteome‐wide recombinant proteins.^14^ The HuProt array is a human proteome microarray with more than 80% coverage of the human proteome to screen for novel biomarkers. In recent years, multiple novel autoantibody candidates in various autoimmune diseases have been identified by this technology.^13^, ^15^

In this study, we performed RA autoantibody screening with HuProt arrays to identify the autoantibody profile of RA patients. After the initial screening and subsequent validation, several autoantibodies were identified as possible diagnostic markers for ACPA‐negative RA, especially in the early stage of disease. A diagnostic model was also constructed to facilitate RA diagnosis.

## 2 RESULTS

### 2.1 Screening of RA‐associated autoantibodies in early RA

**FIGURE 1:**
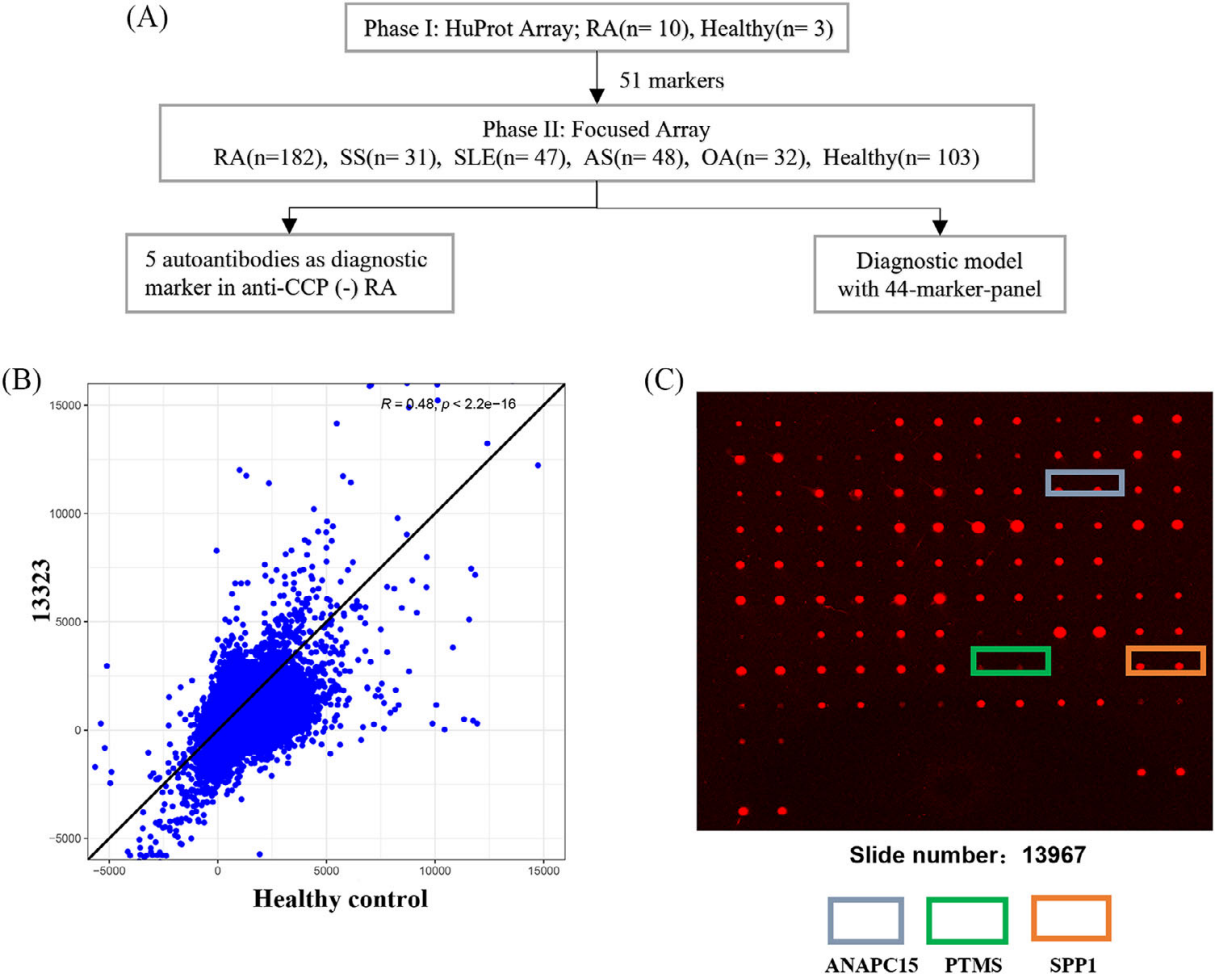
Flow chart of the study design and quality control of HuProt arrays. (A) Flow chart of the study composed of HuProt array in Phase I, customized focused array for validation in Phase II and diagnostic model of 44‐marker‐panel. (B and C) Quality control of HuProt arrays. A total of 10 RA and three control serum samples diluted 1:1000 were incubated with the human proteome microarray. Then, Cy5‐conjugated anti‐human IgG antibody were added. The colored box indicates positive autoantigens. (B) Consistency and diversity autoantibody profiling between healthy control and RA samples. (C) Chip showed that RA sera were recognized with NM_014042 (gray rectangle), NM_002824 (green rectangle), and BC093033 (orange rectangle) respectively.

A two‐phase strategy to screen RA‐associated autoantibodies was applied as previously described^16^ and summarized in Figure 1. In Phase I, we recruited 10 RA patients in the early disease stage (disease duration less than 2 years). Fifty percents (*n* = 5) of these patients were ACPA negative (Table 1). Sera from these RA patients were prescreened on the high content proteome microarrays containing 19,275 human proteins (HuProt™; CDI Laboratories, USA), and sera from healthy donors were applied as controls. The quality of the microarray was further confirmed using the mouse anti‐GST antibody (primary) and a Cy3‐labeled goat‐anti‐mouse IgG antibody (secondary) (Figure S1). Ninety percent of the proteins (about 18,950) on the quality control array had fluorescence intensity ranged between 1000 and 24,000 (Figure S1A). At the same time, 90% of all proteins have a relative percentage difference value less than 30% between duplicates spots (Figure S1B). Autoantibodies against 51 RA‐associated candidate autoantigens were identified (Table S1).

**TABLE 1:** Characteristics of study participants in the study.

	RA—focused array (*n* = 182)		RA—HuProt array	HC	Disease control
	Anti‐CCP+ (*n* = 134)	Anti‐CCP− (*n* = 48)	(*n* = 10)	*n* = 106	*n* = 158
Ages	60.3 ± 13.8	59.0 ± 17.8	53.0 ± 9.9	34.8 ± 9.6	42.4 ± 15.5
Gender (F%)	82.4	75	80	25.6	69.4
Disease duration (y)	11.6 ± 27.8	9.5 ± 10.6	0.6 ± 0.3	–	–
ESR	53.7 ± 33.1	41.0 ± 27.8	61.0 ± 34.5	–	–
CRP	34.3 ± 40.3	41.7 ± 44.9	40.9 ± 34.0	–	–
SJC	6.8 ± 8.6	9.2 ± 9.8	–	–	–
TJC	8.2 ± 9.0	7.5 ± 9.1	–	–	–
DAS28	4.3 ± 1.5	4.4 ± 1.5	–	–	–
Anti‐CCP+ (%)	–	–	50.0	–	–
Smoker%	23.6	13.9	–	–	–
Family RA history%	8.9	5.6	–	–	–
With ILD%	18.7	25.0	–	–	–
Rheumatoid node%	7.3	5.6	–	–	–
SHS (total)	74.3 ± 51.8	78.93 ± 46.2	–	–	–

Data are depicted as median ± SD.

Abbreviations: RA, rheumatoid arthritis; HC, healthy control; DC, disease control; CCP, cyclic citrullinated peptide; RF, rheumatoid factor; ESR, erythrocyte sedimentation rate; CRP, C‐reactive protein; TJC, tender joint count; SJC, swollen joint count; DAS 28, disease activity score 28; ILD, interstitial lung disease; SHS, Sharp score.

**FIGURE 2:**
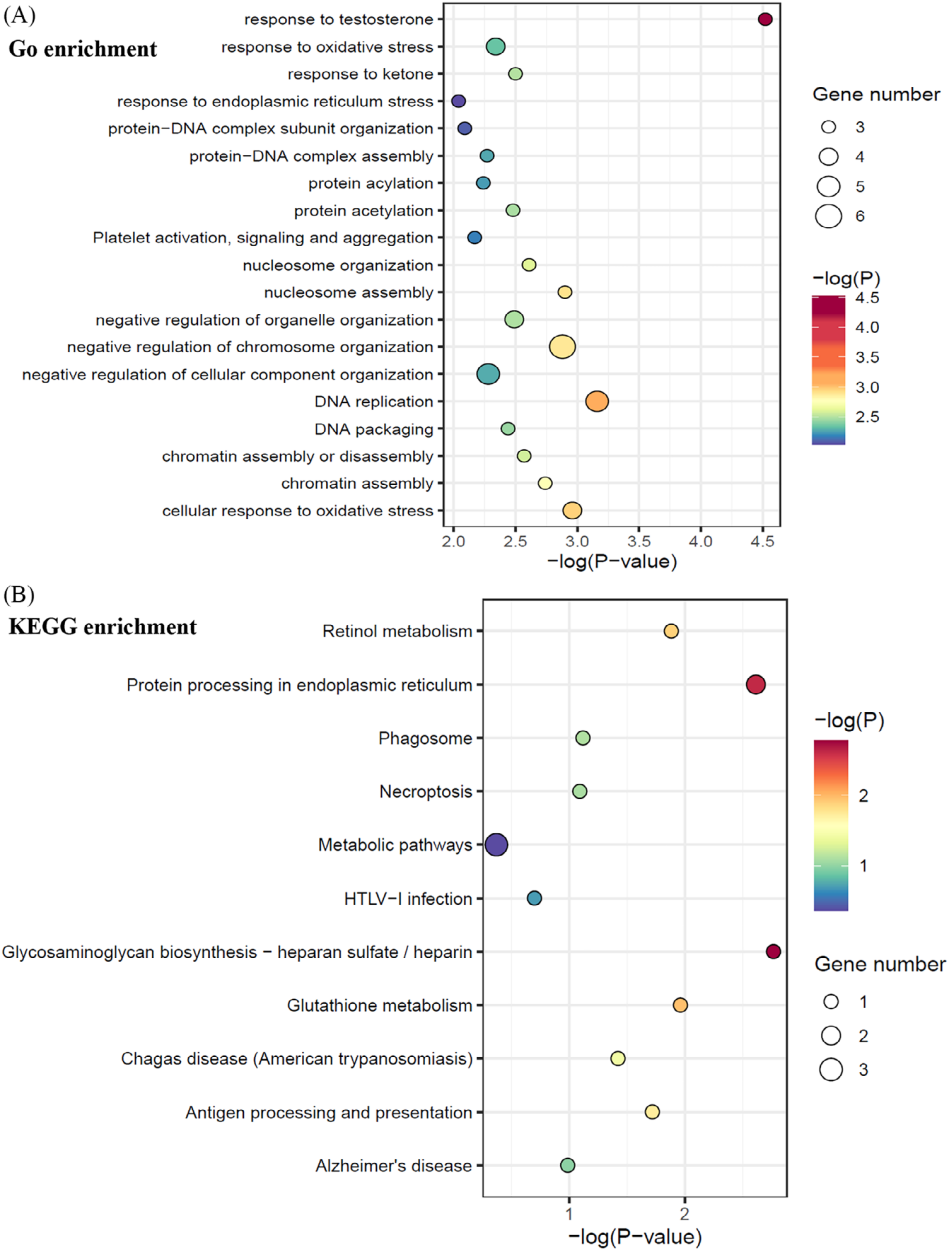
The Kyoto Encyclopedia of Genes and Genomes (KEGG) pathway analysis and GO enrichment analysis of 51 selected candidates of Huprot™ 3.1 Chip. (A) Gene Ontology (GO enrichment analysis). (B) KEGG, Kyoto Encyclopedia of Genes and Genomes.

To clarify the potential involved biological pathways of the 51 autoantibodies, functional enrichment analysis was performed with the antigens against by these autoantibodies (Figure 2A,B). The most significantly enriched GO terms were “response to testosterone” and “DNA replication” (Figure 2A), while KEGG pathways were “Glycosaminoglycan biosynthesis—heparan sulfate / heparin” and “protein processing in endoplasmic reticulum” (Figure 2B) implicating identified RA autoantibodies might contribute to RA pathogenesis via intervening with these biological processes.

### 2.2 Validation of autoantibodies as RA diagnostic markers

In Phase II study, the 51 selected autoantigens were included in a customized focused protein array for further validation with enlarged RA patient cohort and controls. The validation cohort included 182 RA patients and 261 controls (103 heathy controls and 158 disease controls) (Table 1). Area‐under‐the‐curve (AUC) value between 0.7 and 0.9 is regarded to be of good discriminatory power. The AUC of 22 autoantibodies detected by focused protein array were more than 0.7 (Table 2). Among these, anti‐CHAC2 (NM_001008708), anti‐ANAPC15 (NM_014042), anti‐GDE1 (NM_016641), anti‐TSR2 (NM_058163), anti‐CCDC32 (NM_052849), anti‐RDH16 (NM_003708), and anti‐EXTL3 (NM_001440) all showed diagnostic specificity higher than 90%. Fluorescence intensity of the autoantibodies in Table 2 were compared. As shown in Figure S2, box plot analysis showed the autoantibodies in RA were higher than controls (both disease control and healthy control). Anti‐ANAPC15 antibody (NM_014042) was identified as the most specific RA diagnostic biomarker (sensitivity of 41.8%, specificity of 91.5%, AUC of 0.788; Table 2).

**TABLE 2:** Candidate autoantibodies screened by focused array in 182 RA.

				Sensitivity	Specificity	Anti‐CCP (−) (*n* = 48)	
Auto‐antigens		AUC	(95%CI)	%	%	*n*	%
ANAPC15	(NM_014042)	0.788	(0.743,0.832)	41.8	91.5	10	20.8
SPP1	(BC093033.1)	0.785	(0.737,0.833)	52.8	87.7	8	16.7
APBB1	(NM_145689)	0.785	(0.743,0.827)	46.2	87.7	13	27.1
RBBP5	(BC037284.2)	0.779	(0.736,0.823)	45	88.1	9	18.8
MAF1	(BC031273.1)	0.77	(0.727,0.814)	41.8	88.9	7	14.6
LSP1	(BC001785.1)	0.769	(0.725,0.814)	41.8	88.9	10	20.8
TSR2	(NM_058163)	0.755	(0.710,0.800)	35.7	91.2	6	12.5
UBL7	(NM_032907)	0.753	(0.708,0.798)	44	87.3	11	22.9
ILRUN	(NM_024294)	0.744	(0.699,0.790)	36.8	86.9	6	12.5
RDH16	(NM_003708)	0.742	(0.697,0.787)	31.9	90	5	10.4
ZSCAN2	(NM_001007072)	0.741	(0.696,0.787)	35.2	89.2	4	8.3
EXTL3	(NM_001440)	0.735	(0.690,0.781)	28.6	90	3	6.3
BCCIP	(NM_078468)	0.735	(0.690,0.781)	36.3	85.4	9	18.8
GAGE10	(NM_001098413)	0.725	(0.679,0.772)	34.1	88.5	7	14.6
C11orf58	(NM_014267)	0.723	(0.676,0.770)	28.6	88.5	2	4.2
CHAC2	(NM_001008708)	0.718	(0.669,0.766)	12.6	95	5	10.4
RWDD1	(NM_015952)	0.716	(0.668,0.764)	39	85.8	4	8.3
CCDC32	(NM_052849)	0.714	(0.667,0.761)	23.6	90.8	3	6.3
GDE1	(NM_016641)	0.713	(0.666,0.760)	25.3	91.5	2	4.2
PTMS	(NM_002824)	0.71	(0.660,0.760)	39.6	87.7	12	25
AAMP	(NM_001087)	0.703	(0.661,0.756)	28.6	89.6	2	4.2
SH3BGR	(NM_001001713)	0.703	(0.654,0.751)	55.5	74.2	16	33.3

**FIGURE 3:**
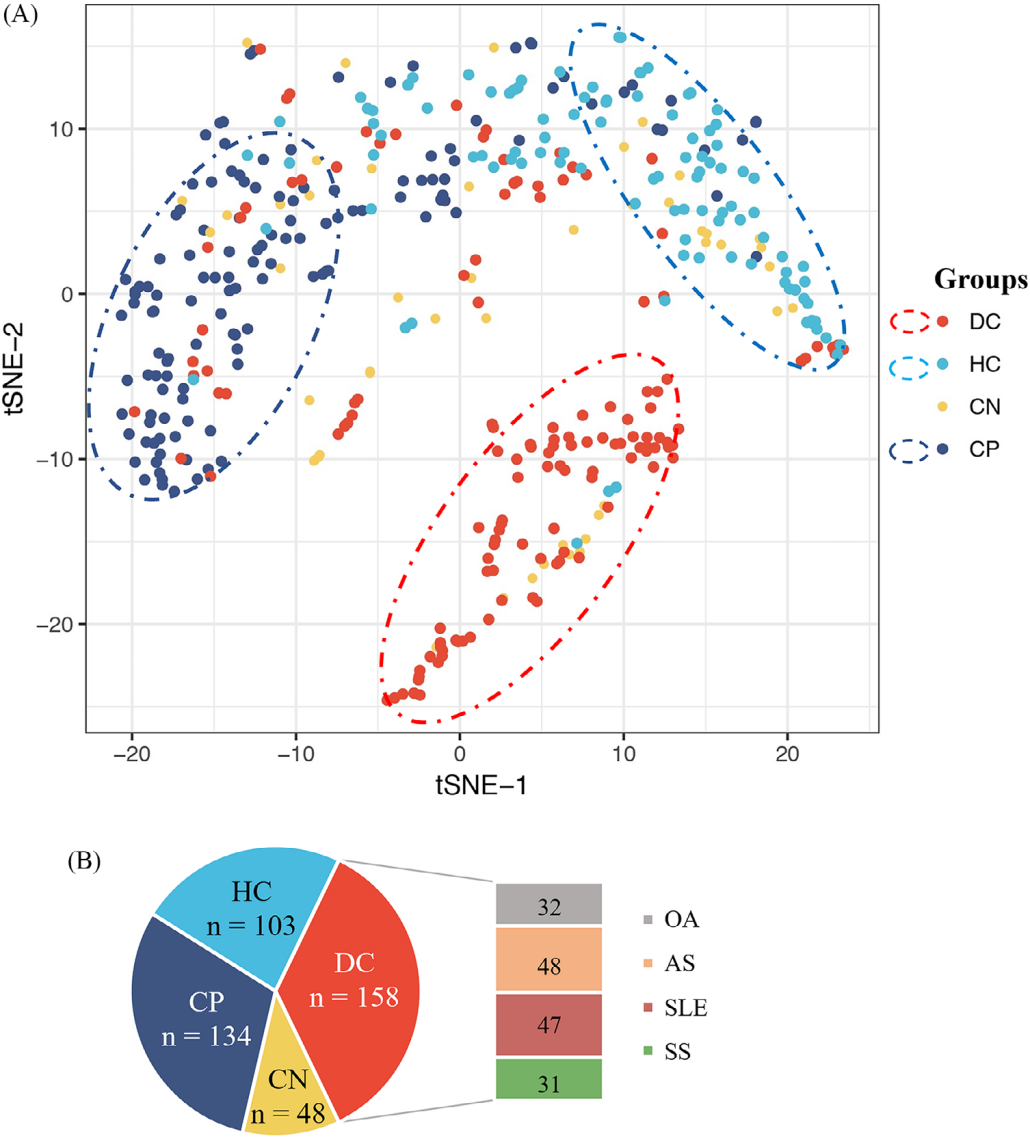
Dot plot for four distinct patient clusters by t‐SNE algorithm based on 51 autoantibodies screened by focused array. (A) Each plot represents one patient, there were 51 dimensions representing 51 autoantibodies, which were reduced to two dimensions (t‐SNE1 and t‐SNE2) by t‐SNE analysis. There were four clusters, red and light blue dots indicate disease control and healthy control. Yellow dots represent anti‐CCP‐negative RA and dark blue represent anti‐CCP‐positive RA. (B) Constitution of patients and controls. DC, disease control; HC, healthy control; CN, anti‐CCP‐negative RA; CP, anti‐CCP‐positive RA; SLE, systemic lupus erythematosus; SS, Sjögren's syndrome; AS, Ankylosing spondylitis; OA, osteoarthritis.

To investigate the expression profiling of the 51 autoantibodies in different disease status, we categorized patients into four groups including ACPA‐positive RA, ACPA‐negative RA, healthy control, and disease control. As shown in Figure 3, the distribution of autoantibodies varies among the four patient groups and clustering into two major modules, indicating genes related to RA through distinct functional mechanisms. Dimension reduction for these 51 autoantibodies by t‐distributed stochastic neighbor embedding (t‐SNE) also showed distinguished patterns characterized by different disease status (Figure 3). Unlike the ACPA‐positive RA patients and the controls, the distribution of ACPA‐negative RA overlapped with the other three groups, indicating that the autoantibody profiles in ACPA‐negative RA were heterogeneous and different from ACPA‐positive RA. Therefore, it was difficult to identify specific autoantibody biomarkers for ACPA‐negative RA diagnosis.

### 2.3 Prevalence of autoantibodies in ACPA‐negative early‐stage RA patients

Prevalence of these newly identified autoantibodies were further examined in ACPA‐negative RA. Five autoantibodies showed sensitivity more than 20% and specificity more than 85% in ACPA‐negative RA (*n* = 48; Table 2), including anti‐ANAPC15 (NM_014042, sensitivity 20.8%, specificity 91.5%), anti‐lymphocyte‐specific protein 1 (LSP1) (BC001785.1, sensitivity 20.8%, specificity 88.9%), anti‐APBB1 (NM_145689, sensitivity 27.1%, specificity 87.7%), anti‐parathymosin (anti‐PTMS, NM_002824, sensitivity 25.0%, specificity 87.7%), and anti‐UBL7 (NM_032907, sensitivity 22.9%, specificity 87.3%). Both sensitivity and specificity of these five autoantibodies were also high in the entire RA cohort (*n* = 182; Table 2). Although prevalence of these autoantibodies in ACPA‐negative RA were not as high as ACPA‐positive RA, they might still be helpful in ACPA‐negative RA diagnosis.

**FIGURE 4:**
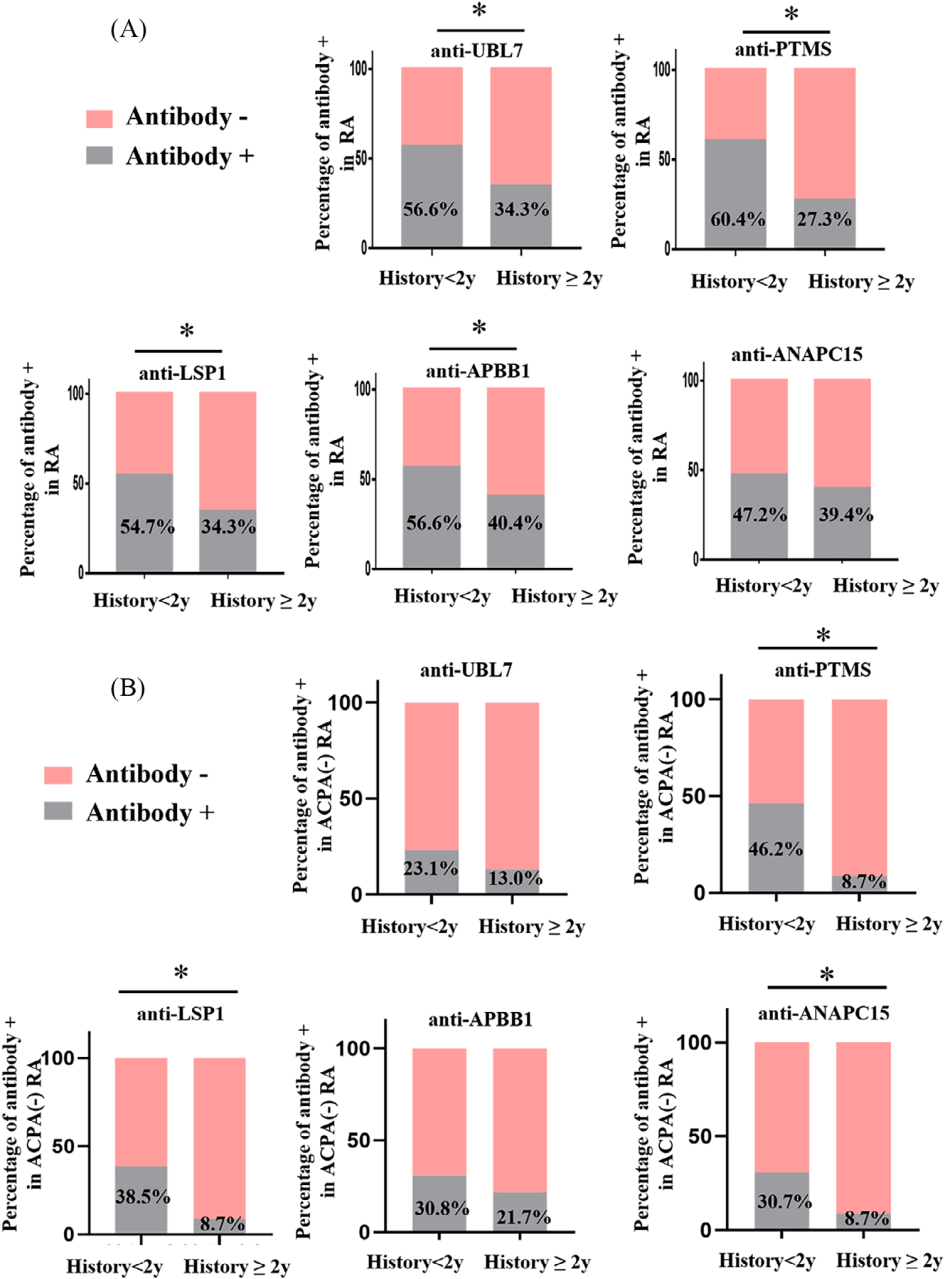
Prevalence of autoantibodies in early stage ACPA‐negative RA. (A) Presence of autoantibodies in early RA. Prevalence based on the cut‐off value of fluorescence signal intensity for five autoantibodies in early RA (history < 2 years, *n* = 53) and established RA (history ≥ 2 years, *n* = 99) were displayed. Gray bars: percentage of autoantibodies‐positive patients; pink bars: percentage of autoantibodies‐negative patients (chi‐square or fisher's exact, *p < 0.05). (B) Presence of autoantibodies in early and ACPA‐negative RA. Prevalence of the five autoantibodies in early RA (history < 2 years, *n* = 13) and established RA (history ≥ 2 years, *n* = 35) with anti‐CCP negative. Gray bars: percentage of autoantibodies‐positive patients; pink bars: percentage of autoantibodies‐negative patients (chi‐square or fisher's exact, *p < 0.05). NM_014042 (anti‐ANAPC15), BC001785.1 (anti‐LSP1), NM_145689 (anti‐APBB1), NM_002824 (anti‐PTMS), and NM_032907 (anti‐UBL7).

We then investigated the five autoantibodies in early RA (duration <2 years, *n* = 53). As shown in Figure 4A, presence of the five autoantibodies were more frequent in early‐stage RA (47.2% positive in anti‐ANAPC15, 54.7% positive in anti‐LSP1, 60.4% positive in anti‐PTMS, 56.6% positive in anti‐APBB1, and 56.6% positive in anti‐UBL7; *p* = 0.3548, 0.0439, 0.0094, 0.0281, and 0.0081, respectively; Figure 4A), compared with established stage (duration ≥2 years, *n* = 99) RA. In particular, it was difficult to diagnose ACPA‐negative RA in early stage. Therefore, we investigated the presence of the five autoantibodies in early stage and ACPA‐negative RA (Figure 4B). In ACPA‐negative RA patients (*n* = 48), there were more autoantibody‐positive patients in early stage (ranging from 21.7 to 46.2%; Figure 4B) than in the established stage (ranging from 8.7 to 21.7%; Figure 4B). Prevalence of antiparathymosin (anti‐PTMS) was the highest in early stage ACPA‐negative RA (46.2%, *p* = 0.016; Figure 4B). Therefore, antiparathymosin (anti‐PTMS) might be helpful in early stage ACPA‐negative RA diagnosis.

### 2.4 Clinical relevance of ACPA‐negative RA‐associated autoantibodies

**FIGURE 5:**
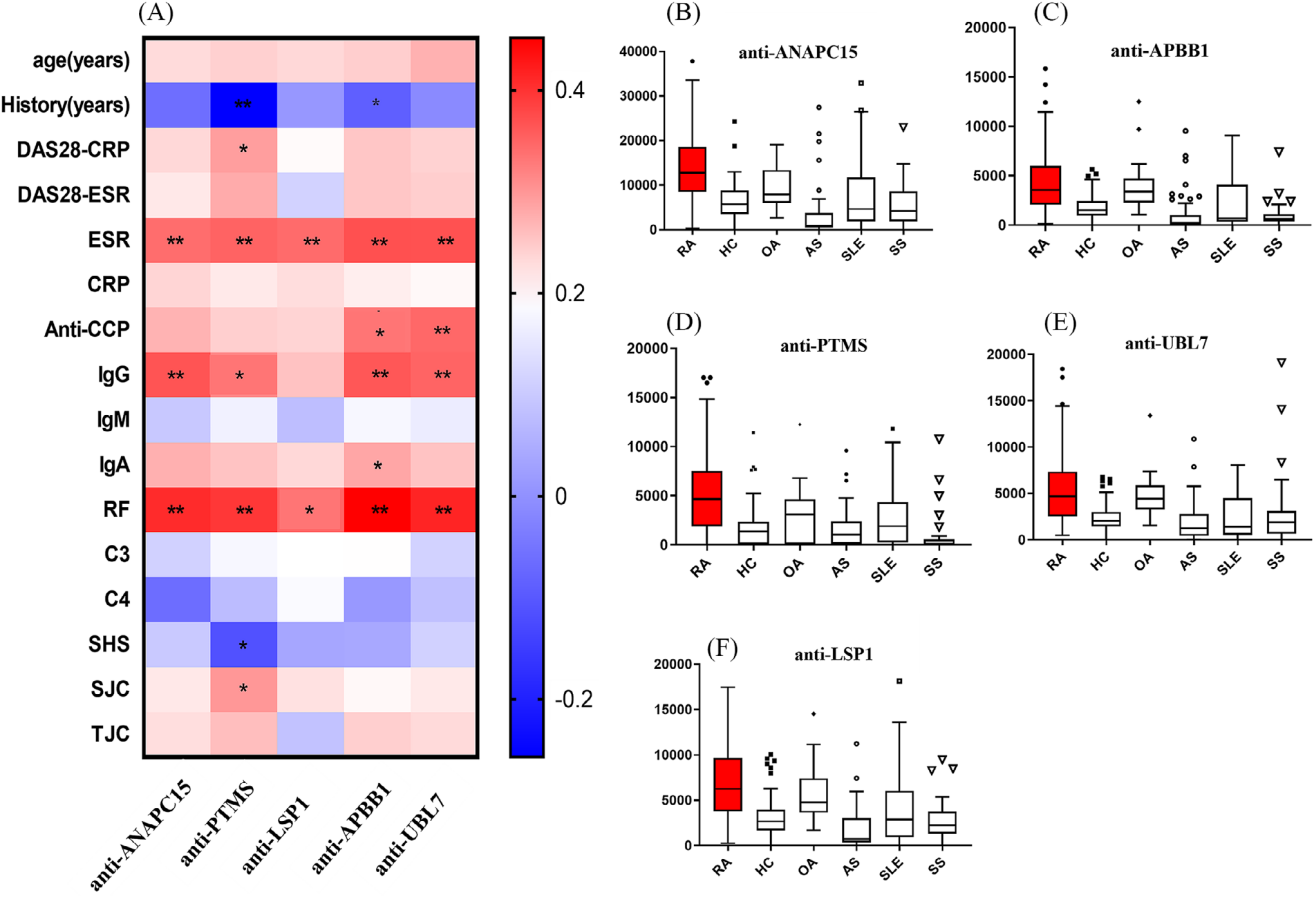
Correlation of diagnostic autoantibodies and clinical significance. (A) Heat map indicating correlations between autoantibodies and immunological characteristics in RA (*n* = 152). Scale color of the filled squares indicates the strength of the correlation (*r*) and whether it is negative (blue) or positive (red). Spearman's rank correlation test or Pearson correlation test. **p < 0.05 and corrected *p* < 0.05; *p < 0.05 and corrected *p* > 0.05, The corrected *p* value is the *p* value calculated after a Bonferroni correction; five autoantibodies with specificity more than 20% in anti‐CCP‐negative RA are: anti‐ANAPC15 (NM_014042), anti‐LSP1 (BC001785.1), anti‐APBB1 (NM_145689), anti‐PTMS (NM_002824), and anti‐UBL7 (NM_032907); SHS, modified Sharp‐van der Heijde Score. (B–F) Fluorescence signal detected for the five autoantibodies mentioned above. The rectangles indicate the interquartile range, and the bar within the rectangle indicates the median value, SEM was showed as error bars. RA, rheumatoid arthritis; HC, healthy controls; OA, osteoarthritis; SLE, systemic lupus erythematosus; CTD, connected tissue disease; AS, Ankylosing spondylitis.

Clinical significance of the five autoantibodies with high specificity for ACPA‐negative RA were analyzed and summarized in Figure 5. The result showed that all of the five autoantibodies positively correlated with erythrocyte sedimentation rate (ESR), IgG, and rheumatoid factor (RF) in RA (*n* = 152). Anti‐parathymosin (NM_002824) was also correlated with disease activity score 28 (DAS28) and swollen joint counts (Figure 5; *r* = 0.170, *p* = 0.031 and *r* = 0.184, *p* = 0.020, respectively), which suggested that anti‐parathymosin might be involved in the pathogenic process of RA inflammation. Interestingly, there was a negative correlation of anti‐parathymosin and joint radiographic progression (Sharp‐van der Heijde score, *r* = −0.175, *p* = 0.047), which indicated that anti‐parathymosin might play a protective role in RA bone erosion. Bonferroni correction was applied to further identify the clinical significance of these autoantibodies with a stricter standard. Clinical correlations between these autoantibodies and inflammatory markers ESR and RF remained statistically significant, and their associations with IgG and anti‐CCP were also significant (Figure 5A).

### 2.5 Construction of diagnostic model with focused array‐derived autoantibodies

**FIGURE 6:**
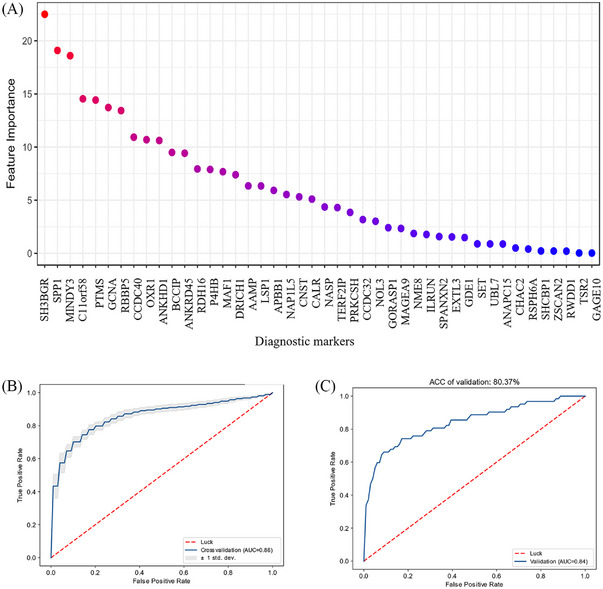
Diagnostic model with focused array‐derived autoantibodies. (A) Feature importance (feature weight *w*) of the 44 selected autoantibodies in SVM model. Feature selection with support vector machine (SVM) model. According to feature weight, 44 autoantibodies were selected. (B) ROC curve of RA diagnosis prediction in cross validation with training data. (C) ROC curve of RA diagnosis prediction with testing data. (B and C) training set (*n* = 240, 120/120) and testing set (*n* = 203, 62/141). Performance of diagnostic model on training set (fourfold cross validation) and testing set, AUROC = 0.86 and 0.84, respectively.

Based on the 51 autoantibodies validated as possible diagnostic markers, we built a quantitative diagnosis model with autoantibodies. Using data‐driven feature selection approach, we identified 44 autoantibodies (Figure 6A) as the final markers to build support vector machine (SVM) model for RA diagnosis. We randomly split the cases into training set (*n* = 240, 120/120, case/control) and testing set (*n* = 203, 62/141, case/control). The model showed high accuracy in cross validation (AUROC = 0.86; Figure 6B). So we built the final model with the whole training set and observed accuracy of 100% in self‐prediction (Figure S3A). With independent testing set, the AUROC is 0.84 (Figure 6C), suggesting its robustness and efficacy in diagnosis prediction. To avoid false positive calls in diagnosis, we increased the threshold for RA calling (specificity = 90% and sensitivity = 67.6% in cross validation). Thus, in the testing set, we observed specificity of 90.8% and sensitivity of 66.1%, which showed significant improvement over using one single autoantibody as the diagnostic marker for RA (Table 2). In ACPA‐negative RA, true positive rate of the prediction model was 23.8% (five out of 21) in cross validation, indicating that the model might also be meaningful to facilitate the diagnosis of ACPA‐negative RA patients.

CH‐28 (CEP‐1/Eno5‐21) and NH‐26 (FIBβ36‐52) are two antibody indicators currently used in clinical practice^17^ and were used as positive controls in focused arrays. Compared with these two indicators applied in clinical practice, the model showed superiority in diagnosis accuracy (Figure S3B). With the specificity at 90%, our model reduced false negative calls significantly (sensitivities: SVM model = 0.66, CH‐28 = 0.40, NH‐26 = 0.22, and CH‐28 + NH‐26 = 0.44).

## 3 DISCUSSION

In the past decades, proteomic techniques including mass spectrometry and protein arrays have been applied in the discovery of novel biomarkers. However, insufficiency of serum biomarkers was still a challenge to RA diagnosis. Both autoantibodies against citrullinated or carbamylated protein modifications have been proved to be of diagnostic and prognostic significance.^10^, ^11^ However, autoantibodies against other proteins are also abundant in RA patients.^18^ The profiles and clinical significance of these autoantibodies are not fully elucidated. Therefore, we screen novel autoantibodies with genome‐wide arrays to explore more details in RA.

ACPAs have been elucidated to be associated with bone erosion and joint destruction. Genetic studies also indicated that ACPA‐positive RA and ACPA‐negative RA are two distinct disease entities. Diagnosis for ACPA‐negative RA was difficult due to lack of specific markers, especially in early stage.

In the present study, we identified five novel autoantibodies with possible diagnostic value in ACPA‐negative RA (anti‐ANAPC15, anti‐LSP1, anti‐APBB1, anti‐PTMS, and anti‐UBL7), especially in the early stage. It was interesting that there was 46.2% antiparathymosin positive in early ACPA‐negative RA, making antiparathymosin a possible biomarker for early ACPA‐negative RA. However, further validation in larger early ACPA‐negative RA cohorts is in need in future studies.

Based on KEGG annotation, identified antibodies significantly enriched in protein processing in ER and metabolic pathway, indicating that the two pathways play important roles in autoimmune response in RA pathogenesis. Protein processing in ER, include calreticulin (CRT; also called CALR) and PDIs (also called P4HB; see Figure S4A), which participated in correct, misfolded protein process and ubiquitylation (Figure S4A). CRT (CALR) may also be enriched in antigen presentation pathway (Figure S4B) and exert an effect in MHC class I molecule assemble pathway. Therefore, CRT (CALR) seems to be a common mediator in “ER protein processing” pathway. Further functional study of CRT needs to be carried out.

Metabolic pathways including glutathione and retinol metabolism were also involved in our pathway enrichment analysis, which indicated that RA autoantibodies were involved in the metabolism regulation. Glutathione‐specific gamma‐glutamylcyclotransferase was enriched in our KEGG analysis (Figure S4C), but it has not been reported in RA previously. However, evidence showed that oxidative stress and antioxidants system might play crucial roles in RA.^19^ Glutathione and its related metabolic enzymes were important member in oxidative stress, which were reported to be diagnostic markers or disease activity indicators in RA.^20^, ^21^ Therefore, autoantibodies targeting glutathione related enzyme may affect RA pathogenesis through oxidative stress. Functional study of autoantibodies targeting glutathione metabolic pathway will be carried out in our future work. As shown in Figure S4D, retinol dehydrogenase 8 (RDH) in retinol metabolism pathway was also enriched in our KEGG analysis. Although RDH has not been reported in RA, retinol acid metabolism has been shown in RA.^22^, ^23^, ^24^ Therefore, RDH autoantibody may affected RA through retinol acid metabolic pathway.

Autoantibody against cartilage oligomeric matrix protein (COMP; BC033676) has been reported previously as a diagnostic marker in RA and was related with disease activity by Ge et al.^25^, ^26^, ^27^ Furthermore, (COMP) fragments derived from cartilage were isolated from synovial fluids of RA.^26^, ^27^ Murine monoclonal anti‐COMP antibody could induce arthritis in naive mice.^25^ Proteins, including proline‐4‐hydroxylase (P4H), LSP1, and CALR, have also been clarified to be functionally related with RA,^28^, ^29^, ^30^, ^31^ but the corresponding autoantibodies have not been identified in RA patients. These results supported the reliability of HuProt proteome array in RA‐associated autoantibody screening.

Among the 51 autoantibodies identified in Phase I screening, antiparathymosin was the only one negatively correlated with bone erosion. Parathymosin, also called macromolecular translocation inhibitor II (MTI‐II),^32^ was a Zn^2+^ binding protein and first isolated from bovine thymus gland,^33^, ^34^ acting as a coactivator of glucocorticoid receptors.^35^ Parathymosin was able to inhibit suppression of BMP‐Smad‐induced osteogenic differentiation,^36^ which might explain the protective role in bone erosion. However, the mechanism of antiparathymosin in RA bone destruction still needs further investigation.

In this study, an unbiased genome‐wide protein array‐based approach was employed to survey autoantibodies against the human proteome in RA patients. HuProt array has also been performed by Li et al.^13^ and Auger et al.^37^ in RA patients. A total of 120 sera were applied to HuProt microarray by Li et al^13^. And 35 candidate autoantigens were identified, only two of which were overlapped with our study (LSP1 and CHAC2). In their focused array (290 RA vs. 197 controls), autoantibodies anti‐PTX3 and anti‐DUSP11were identified as biomarkers for anti‐CCP‐negative RA. Auger et al.^37^ also identified autoantibodies by HuProt array in 19 RA versus 23 controls, and their result did not overlap with our study. Since few autoantibodies were common between these independent studies, we cannot validate our model by these datasets. The phenomenon that few autoantibodies overlapped among these independent datasets might be due to different screening strategies, different recruited cohorts and different versions of HuProt arrays. Since an independent replication could not be performed, we need to recruit a larger number of patients in the future studies to validate the reliability and performance of the possible diagnostic value of these newly identified RA‐associated autoantibodies.

In conclusion, our study identified five autoantibodies in ACPA‐negative RA by HuProt array, especially in early‐stage patients. We also constructed a penal with higher positivity in RA than single markers. This autoantibody‐array based strategy might be useful for a particular disease.

## 4 MATERIALS AND METHODS

### 4.1 Patients and controls

Serum samples from 192 RA patients (including 53 anti‐CCP‐negative RA, and 139 anti‐CCP‐positive RA), 47 systemic lupus erythematosus (SLE) patients, 31 Sjögren's syndrome (SS) patients, 48 Ankylosing spondylitis (AS) patients, 32 osteoarthritis (OA) patients, and 106 healthy volunteers were collected between 2015 and 2017 at the Department of Rheumatology and Immunology, Peking University People's Hospital.

RA patients met the 2010 ACR and EULAR criteria for RA, all SLE (SLICC Revision of ACR 2009), SS (ACR 2012), AS (Modified New York criteria 1984), and OA (ACR1995) fulfilled their classification criteria, respectively. The study was approved by the Research Ethics Committee at Peking University People's Hospital, Beijing, China.

All RA serum samples were tested for anti‐CCP detection by the second‐generation ELISA kit (Fuchun‐Zhongnan Biotech Co., Shanghai, China) with a cutoff value of 25 U/mL. ACPA negative was defined as anti‐CCP negative. RF (IgM) level was measured by the Rate nephelometry (Immage; Beckman Coulter, Fullerton, CA, USA); values above 20 U/mL for RF were considered positive.

Radiographs of the hands of patients with RA were studied by two experienced radiologists blinded to patients’ clinical and laboratory data. Radiographs were scored according to the Sharp‐van der Heijde method.^38^, ^39^

### 4.2 Serum profile of human proteome microarrays

The human proteome microarray (HuProt™) V3.1 was composed of about 19,275 full‐length human proteins and was constructed in CDI company.^14^ This microarray contained 48 blocks arranged in a 25 × 32 array layout. Each sample was printed in duplicate, the control probes include printing buffer, human IgG, human‐IgM, biotin‐BSA, BSA, and GST. All of the recombinant human proteins were generated by the *Saccharomyces cerevisiae* expression system and carried an N‐terminal GST tag.^40^ The quality of the microarray was measured by using mouse anti‐GST and anti‐mouse IgG antibody.

A total of 10 RA (five anti‐CCP positive and five anti‐CCP negative) was probed individually to HuProt™ arrays and three healthy donors were used as control. HuProt™ (Human Proteome Microarray v3.1; CDI Labs, Mayaguez, PR), which contains over 19,275 full‐length individually purified human proteins in duplicate, covering more than 75% of the proteome.^14^, ^16^ Briefly, the HuProt™ arrays were blocked with blocking solution (5% BSA/TBS‐T) at room temperature for 1 h, and then probed with serum samples (diluted 1:1000) at 4°C overnight. Arrays were then washed three times with 1× TBS‐T, 10 min each, and probed with Alexa‐647‐labeled anti‐human IgG (Jackson ImmunoResearch, West Grove, PA) at room temperature for 1 h, followed by three washes of 1× TBS‐T, 10 min each, and then spun to dryness prior to scanning. The slides were scanned on a GenePix 4200A microarray scanner (Axon Instruments) and the raw Genepix Array List file was aligned. To identify positive hits on HuProt™ arrays, both spots of protein printed in duplicate had to be present in samples but not in a blank or secondary antibody‐only control.

### 4.3 Construction of RA focused microarray

A total of 51 candidate autoantigens were chosen and subjected to sub‐arrays on a single OPEpoxy Slide™ to construct the RA sub‐microarrays. Selection criteria was that the signal noise ratio of each protein was higher than 13 and that it had net fluorescent signal intensity (F532‐B532) more than 2.5 times of that of the pooled control samples. In Phase II, 182 RA patients and 261 controls (103 heathy control and 158 disease control including 48 AS, 47 SLE, 31 SS, 32 OA) were subjected to the RA sub‐array profiling. The protein microarray experiment was carried out in accordance with the protocol provided by the manufacturer. CH‐28 (CEP‐1/Eno5‐21 from α‐enolase) and NH‐26 (FIBβ36‐52 from fibrinogen) are citrullinated peptides that were recognized by ACPA (anticitrullinated protein antibodies) from RA patients. CH‐28 and NH‐26 were positive controls in the focused array.

### 4.4 RA prediction model derived from focused array

For the 51 candidate autoantigens measured by focused array, we built SVM model with radial basis function (RBF) kernel and optimized the best parameters according to grid search (*C* = 32, *γ* = 2; Figure S5). Then, we adopted recursive feature elimination (RFE) for feature selection and used the final set of 44 autoantigens to build the model (Figure 6A). The RFE for SVM with RBF kernel and feature weight *w* ^2^ calculation was implemented following the approach introduced by Liu et al.^41^ with the package “scikit‐learn” in python.^42^ To reduce false positive calls, we set the score at specificity of 90% in training set as the threshold for RA diagnosis. We provided the model and script for RA prediction with human protein array at https://github.com/gao‐lab/RA_prediction.

### 4.5 Statistics

All statistical analysis was performed with GraphPad Prism 8 (GraphPad Software Inc., San Diego, CA) and SPSS 17.0 software. Differences between various groups were evaluated by the Student's *t*‐test, Mann–Whitney *U* test, Chi‐square test, or Spearman's rank correlation test. The receiver‐operating characteristic curve analysis was performed with the statistical software SPSS 17.0 to evaluate the diagnostic utility of autoantibodies using nonparametric method. The optimal positive cut‐off value in the study was set for 2 SD above the mean value of the healthy controls. All statistical analyses with *p* value < 0.05 were considered statistically significant (*p < 0.05, **p < 0.01, ***p < 0.001, N.S., not significant).

## AUTHOR CONTRIBUTIONS

Xu Liu, Zhanguo Li, and Xiaolin Sun contributed to the conception and design of the study. Ge Gao designed and guided the RA prediction model construction. Xiaoying Zhang and Xu Liu participated in experiments data collection and clinical analysis. Xu Liu and Yujiang Kang analyzed the data. Xu Liu, Yujiang Kang, Zhanguo Li, and Xiaolin Sun drafted the manuscript. Fei huang, Shuang Liu, Yixue Guo, Yingni Li, Changcheng Yin, Jiahe Li, Wangzha Pingcuo, and Yan Zhang helped the data analysis and the manuscript edit. Mingling Liu, Qimao Han, Qingwen Wang, Jiahe Li, Wangzha Pingcuo, and Yan Zhang helped in clinical data collection. Hua Ye, Haihong Yao, Chun Li, Jiahe Li, Wangzha Pingcuo, and Yan Zhang helped to prepare for figures and tables. All authors read and approved the final manuscript and consented to publish this manuscript.

## CONFLICT OF INTEREST STATEMENT

The authors declare that they have no conflict of interests that could be perceived as prejudicing the impartiality of the research reported.

Author Changcheng Yin is an employee in Beijing Protein Innovation Co. Ltd, but has no potential relevant financial or nonfinancial interests to disclose. The other authors have no conflicts of interest to declare.

## ETHICS STATEMENT

Human specimens: RA patients, disease control patients, and healthy donors’ blood sample acquisitions were approved by the Ethics Committee of Peking University People's Hospital with approval number of 2020PHB036‐01. Written informed consent has been obtained from all patients.

## Supporting information

Supporting Information

## Data Availability

All data are available from the corresponding authors upon request.
