# Supplementary material for: An autoantibody profile identified by human genome‐wide protein arrays in rheumatoid arthritis
Source: MedComm (2020). 2024 Aug 11;5(8):e679. doi: 10.1002/mco2.679 (PMC11317183; doi:10.1002/mco2.679)
Supplement: Supplementary file 1 — Supporting Information [file MCO2-5-e679-s001.docx]

**An autoantibody profile identified by human genome-wide protein arrays in rheumatoid arthritis**

Xu Liu^1†^, Xiaoying Zhang^1†^, Yu-Jian Kang^2†^, Fei Huang^4^, Shuang Liu^5^, Yixue Guo^1^, Yingni Li^1^, Changcheng Yin^6^, Mingling Liu^7^, Qimao Han^8^, Qingwen Wang^9^, Hua Ye^1^, Haihong Yao^1^, Chun Li^1^, Jiahe Li^1^, Wangzha Pingcuo^1^, Yan Zhang^1^, Yin Su^1^, Ge Gao^3*^, Zhanguo Li^1*^, Xiaolin Sun^1*^

Running head：Genome-wide screening of autoantibody profiles in RA

1. Department of Rheumatology and Immunology, Peking University People’s Hospital & Beijing Key Laboratory for Rheumatism Mechanism and Immune Diagnosis (BZ0135), Beijing, China.

2. Chongqing Key Laboratory of Intelligent Oncology for Breast Cancer, Cancer Hospital, School of Medicine, Chongqing University, Chongqing 400030, China

3. State Key Laboratory of Protein and Plant Gene Research, School of Life Sciences, Biomedical Pioneering Innovative Center (BIOPIC) & Beijing Advanced Innovation Center for Genomics (ICG), Center for Bioinformatics (CBI), Peking University

4. General medical department, Huazhong University of Science and Technology Union Shenzhen Hospital 518052, China.

5. Department of Rheumatology and Immunology, First Affiliated Hospital of Kunming Medical University. No. 295, Xichang Road, Wuhua District, Kunming, Yunnan Province, 650032.

6. Beijing Protein Innovation，B-8, Airport Industrial Zone, Beijing, China (101318) .

7. Department of Rheumatology, the First Affiliated Hospital of Guangzhou University of Chinese Medicine. Guangzhou China, 510405.

8. Department of Rheumatology, The First Affiliated Hospital of Heilongjiang University of Traditional Chinese Medicine. No.24 Heping Road, Xiangfang District, Harbin, China.

9. Department of Rheumatism and Immunology, Peking University Shenzhen Hospital, Shenzhen, China.

These authors contributed equally to this work and share first authorship: Xu Liu^†^, Xiaoying Zhang^†^, Yu-Jian Kang^†^

*✉email correspondence to: sunxiaolin_sxl@126.com; li99@bjmu.edu.cn; gaog@mail.cbi.pku.edu.cn

**Key words:** rheumatoid arthritis; anti-citrullination protein antibody (ACPA); HuProt array; autoantibody

Supplementary Figures


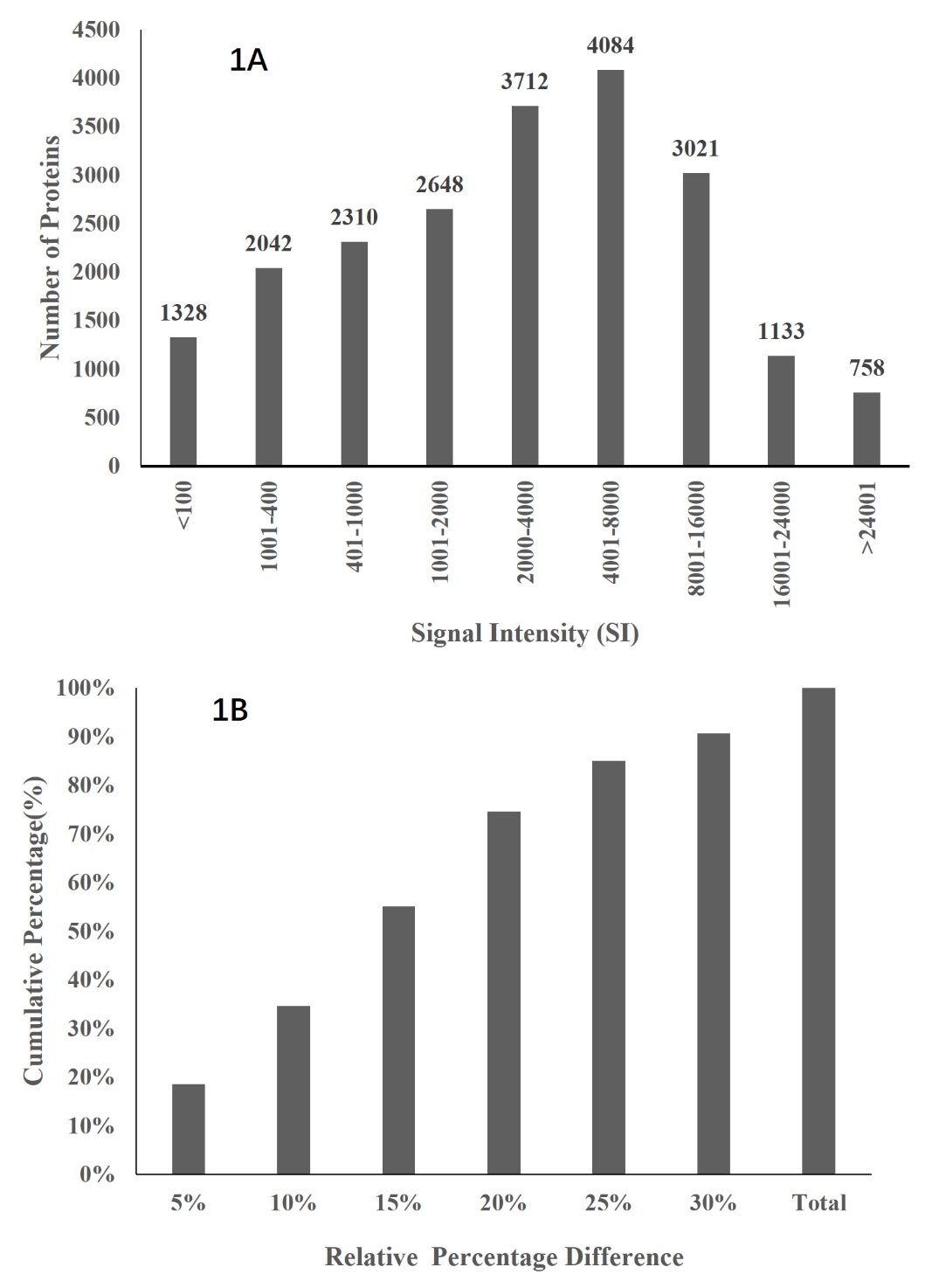


Figure S1. Quality control of HuProt arrays

S1A.Fluorescence intensity on quality control array. S1B. Cumulative variation on quality control array


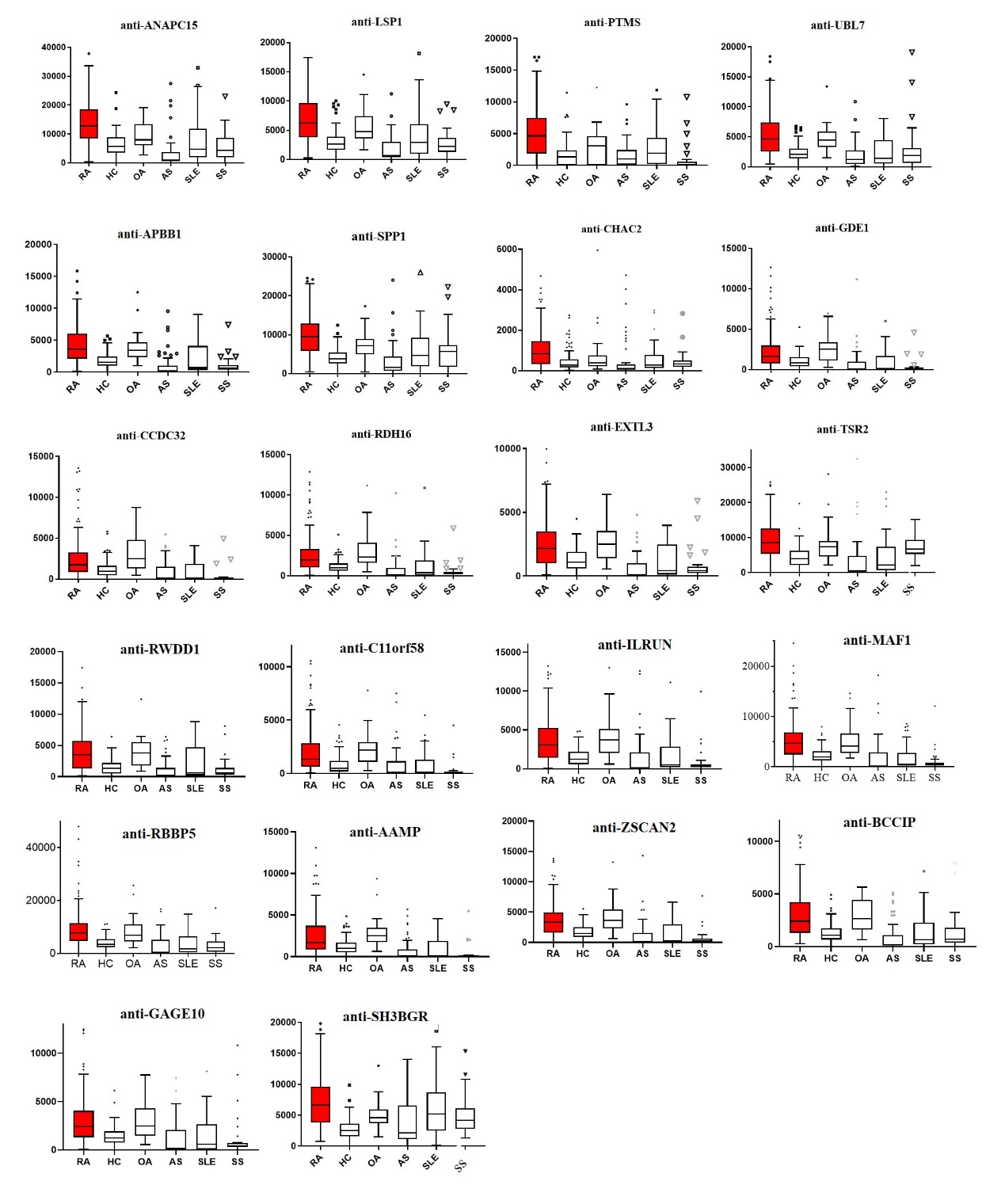


**Figure S2.**  Box plots of the array-identified autoantigens in RA and controls. The fluorescence of newly identified autoantibodies are displayed in box plots. The rectangles indicate the interquartile range, and the bar within the rectangle indicates the median value, SEM was showed as error bars.

RA: Rheumatoid arthritis; HC: healthy controls. OA: Osteoarthritis, SLE: Systemic lupus erythematosus, CTD: connected tissue disease, AS: Ankylosing Spondylitis.


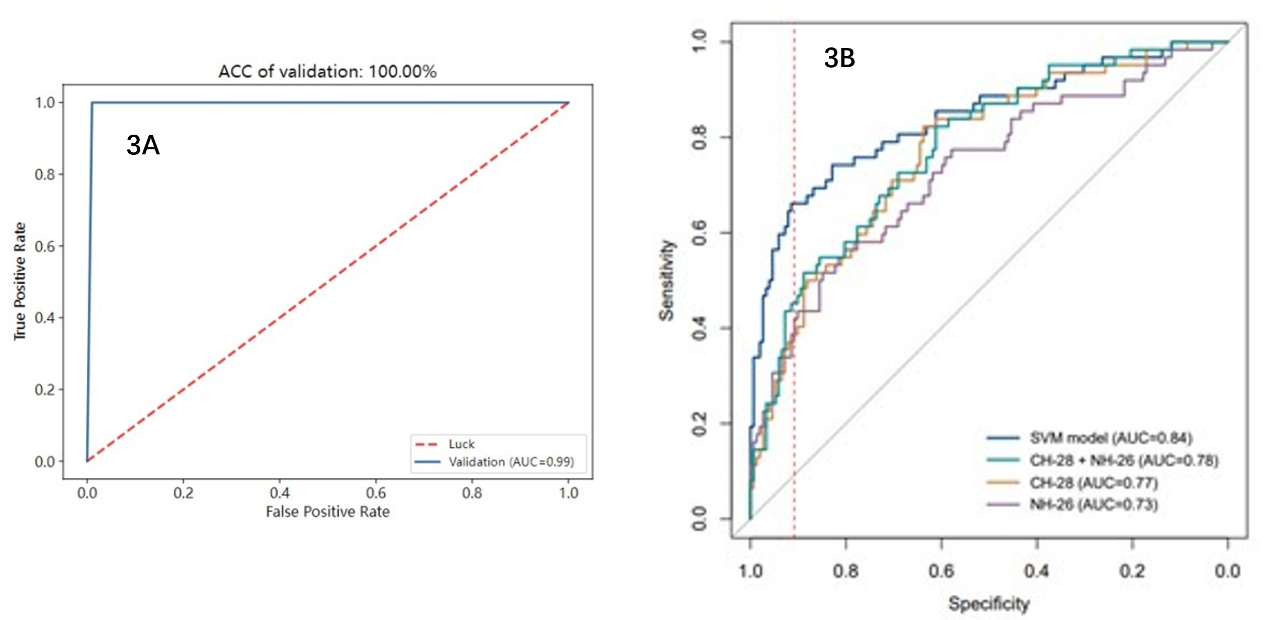
 **Figure S3.** Internal testing and internal validation of the diagnostic model.

S3A. AUCROC curve of self-prediction with training data. S3B. ROC curves of our model, CH-28 and NH-26 in the testing set. “CH-28 + NH-26” refers to linear model built with the two indicators. The red dashed line showed the threshold of the SVM model (specificity of 90.8%).

**
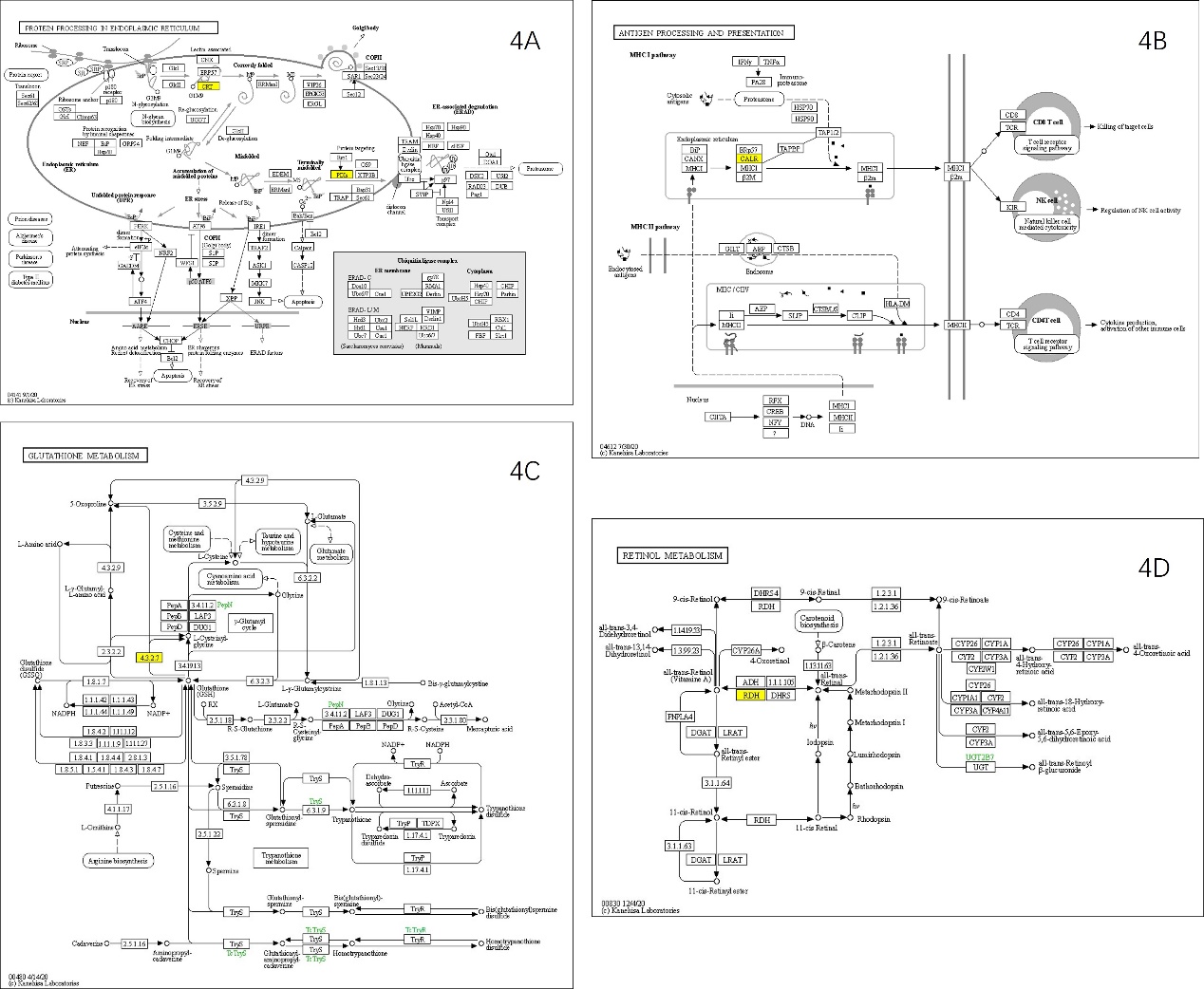
**

Figure S4. Kegg pathways of newly identified autoantigens. S4A: protein processing of endoplasmic reticulum; S4B: antigen processing and presentation; S4C: glutathione metabolism; S4D: retinol metabolism

**
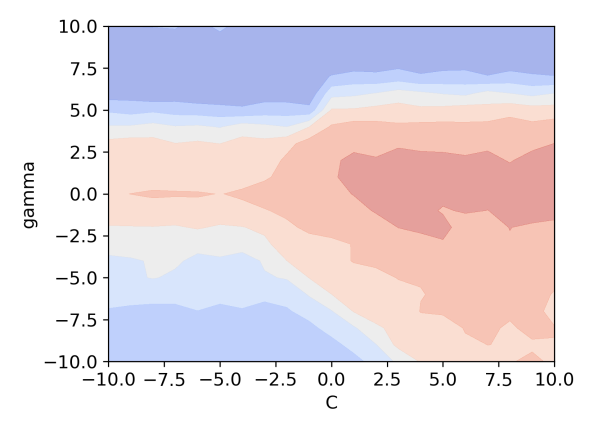
**

**Figure S5.** Grid search for parameter C and gamma (log2 transformed). The darkest red region showed accuracy beyond 0.8.

**Supplementary Tables**

**Table S1.** Name of the 51 candidate autoantigens from Huprot^TM^ focused Array

| Protein ID | Name |
| --- | --- |
| NM_015952 | RWDD1 ([RWD domain containing 1](https://www.ncbi.nlm.nih.gov/nuccore/NM_015952.4/)) |
| NM_014267 | C11orf58 (chromosome 11 open reading frame 58) |
| NM_058163 | TSR2 (TSR2 ribosome maturation factor) |
| BC007200.1 | ANP32A (acidic (leucine-rich) nuclear phosphoprotein 32 family, member A) |
| NM_024294 | ILRUN (inflammation and lipid regulator with UBA-like and NBR1-like domains) |
| NM_007051 | FAF1 (Fas associated factor 1 ) |
| NM_152298 | NASP (nuclear autoantigenic sperm protein ) |
| NM_001008708 | CHAC2 (ChaC glutathione specific gamma-glutamylcyclotransferase 2) |
| NM_012196 | GAGE8 (G antigen 8 ) |
| BC031273.1 | MAF1 (MAF1 homolog (S. cerevisiae)) |
| NM_014042 | ANAPC15 (anaphase promoting complex subunit 15 ) |
| NM_024745 | SHCBP1 (SHC binding and spindle associated 1) |
| NM_016616 | NME8 (NME/NM23 family member 8 ) |
| NM_005365 | MAGEA9 (MAGE family member A9) |
| NM_017978 | ANKHD1 (ankyrin repeat and KH domain containing 1) |
| NM_016449 | DRICH1 (aspartate rich 1) |
| NM_000918 | P4HB (prolyl 4-hydroxylase subunit beta) |
| BC001785.1 | LSP1 (lymphocyte-specific protein 1) |
| BC013586.1 | PRKCSH (protein kinase C substrate 80K-H) |
| NM_145689 | APBB1 (amyloid beta precursor protein binding family B member 1) |
| NM_003946 | NOL3 (nucleolar protein 3) |
| NM_130776 | XAGE3 (X antigen family member 3) |
| NM_182498 | ZNF428 (zinc finger protein 428) |
| ENST00000374876 | CCDC40 (Coiled-coil domain containing 40) |
| NM_052957 | GCNA (germ cell nuclear acidic peptidase) |
| NM_002824 | PTMS (parathymosin) |
| NM_198493 | ANKRD45 (ankyrin repeat domain 45 ) |
| NM_024948 | MINDY3 (MINDY lysine 48 deubiquitinase 3) |
| NM_052849 | CCDC32 (coiled-coil domain containing 32) |
| NM_031899 | GORASP1 (golgi reassembly stacking protein 1) |
| NM_001001713 | SH3BGR (SH3 domain binding glutamate rich protein) |
| NM_153757 | NAP1L5 (nucleosome assembly protein 1 like 5 ) |
| NM_018975 | TERF2IP (TERF2 interacting protein ) |
| BC037284.2 | RBBP5 (retinoblastoma binding protein 5) |
| NM_001087 | AAMP (angio associated migratory cell protein) |
| NM_032907 | UBL7 (ubiquitin like 7) |
| NM_001007072 | ZSCAN2 (zinc finger and SCAN domain containing 2) |
| NM_003011 | SET (SET nuclear proto-oncogene ) |
| BC093033.1 | SPP1 (secreted phosphoprotein 1) |
| BC010228.2 | CNST (chromosome 1 open reading frame 71) |
| NM_001040663 | GAGE1 (G antigen 1) |
| NM_001440 | EXTL3 (exostosin like glycosyltransferase 3) |
| NM_016641 | GDE1 (glycerophosphodiester phosphodiesterase 1) |
| NM_004343 | CALR (calreticulin) |
| NM_078468 | BCCIP (BRCA2 and CDKN1A interacting protein ) |
| AL136761 | RSPH6A (cDNA DKFZp434I0515) |
| BC032710 | OXR1 (oxidation resistance 1) |
| BC033676 | COMP (cartilage oligomeric matrix protein) |
| NM_001009615 | SPANXN2 (SPANX family member N2) |
| NM_003708 | RDH16 (retinol dehydrogenase 16) |
| NM_001098413 | GAGE10 (G antigen 10) |
